# Enhanced omnidirectional and weatherability of Cu_2_ZnSnSe_4_ solar cells with ZnO functional nanorod arrays

**DOI:** 10.1038/s41598-017-14899-4

**Published:** 2017-11-02

**Authors:** Fang-I Lai, Jui-Fu Yang, Wei-Xiang Liao, Shou-Yi Kuo

**Affiliations:** 1 Department of Photonics Engineering, Yuan-Ze University, 135 Yuan-Tung Road, Chung-Li, 320 Taiwan; 2 Advanced Optoelectronic Technology Center, National Cheng-Kung University, Tainan, 701 Taiwan; 3 Department of Electronic Engineering, Chang Gung University, 259 Wen-Hwa 1st Road, Kwei-Shan, Tao-Yuan 333 Taiwan; 4 Department of Nuclear Medicine, Chang Gung Memorial Hospital, Linkou, No. 5, Fuxing Street, Kwei-Shan, Tao-Yuan 333 Taiwan

## Abstract

This paper presents the use of nanorods of different sizes, deposited from a chemical solution, as an antireflection layer in copper–zinc–tin selenide (CZTSe) solar cells. With the aid of the nanorods, the surface reflection of the CZTSe solar cells was reduced from 7.76% to 2.97%, and a cell efficiency of 14% was obtained as a result. Omni-directional anti-reflection was verified by the angle-dependent reflection measurements. The nanorod arrays also provided the CZTSe solar cells with a hydrophobic surface, allowing it to exhibit high resistance against humidity during weatherability tests. This shows that the surface passivation brought by the nanorod layer at the surface could effectively extend the lifetime of the CZTSe solar cells. The rate of efficiency decay of the CZTSe solar cells was reduced by 46.85% from that of the device without a nanorod array at the surface, indicating that this surface layer not only provided effective resistance against reflection at the device surface, but also served as a passivation layer and humidity-resistant surface-protection layer.

## Introduction

As solar cells are being expected to become an effective alternative to the environmentally hazardous fossil fuel that is believed to be depleted in the near future, some solar-cell technologies have been found to be hampered by the materials or methods used during manufacturing. For example, some of the materials are toxic and environmentally unsafe, and the sources of rare-earth materials utilized in the absorber layer are depletable^1^. Solar cells can be divided into two basic categories— bulk and thin-film solar cells. Copper–(indium, gallium) selenide (Cu(In,Ga)Se_2_, CIGS) and other materials in the same family with similar characteristics are the most promising candidates for materials in thin-film solar cells because of their high absorption coefficient, changeable bandgap, and resistance to photo-degradation^2,3^. However, many thin-film solar-cell technologies are hampered by the use of toxic materials, high material consumption, and the use of rare-earth raw materials, leading to very limited production and application, which make widespread adoption of thin-film solar cells economically unrealistic. To overcome this barrier, research and development have been carried out to search for alternative materials that are abundant, non-toxic, low-cost, and suitable for highly efficient solar cells to provide an economically competitive energy source in the near future. Copper–zinc–tin sulfide (Cu_2_ZnSnS_4_, CZTS) solar cells have been successfully developed for this purpose. CZTS is related to the CIGS family that possesses all the characteristics required for photovoltaics, and it also has the merit of replacing the rare-earth elements In and Ga in CIGS with abundant earth elements Zn and Sn.

The antireflection treatment currently used for CZTS and CIGS solar cells is depositing a single antireflection layer on top of the device to provide refractive-index matching and reduce surface reflection. The refractive index of this antireflection layer lies between the refractive indices of air and the top layer of the solar cell. In addition, surface roughening using different nanoscale or microscale structures, which is achieved by wet etching or lithography, is also be employed to reduce light reflection from solar cells^4–6^. However, this method can result in a high concentration of carrier-recombination centres on the etched surface, leading to massive loss of photo-current and low efficiency of the solar cell^7^. Most of the antireflection surface structures were also limited to a small area, and their creation required high fabrication cost. To resolve these problems, large-area surface structures consisting of arrays of ZnO nanorods were fabricated in the current study by a simple, low cost, room-temperature synthesis process. The profile of these surface structures can also be changed by adjusting the synthesis parameters. The array of nanorod surface structures was applied to the CZTSe solar cell in this study. Aluminum-doped ZnO (ZnO:Al, AZO) was selected for the transparent conductive layer on the top of CZTSe solar cell. The synthesis of the layer of ZnO nanorods on top of the AZO layer was carried out as homogeneous material overgrowth. As a result, the ZnO nanorod layer was easier to fabricate and was thermally and mechanically stable owing to the match between the chemical and physical characteristics of the layers. The two layers also share the same optical and electrical properties, such as a direct bandgap of 3.37 eV and high transmittance in the visible spectral range^8–10^, making ZnO a perfect material for antireflection surface structures and the technique the most promising antireflection approach for CZTSe solar cells.

To date, there have been many published studies on the fabrication of nanostructures as the surface antireflection layer in CIGS and CZTSe solar cells^11–13^. Nonetheless, most studies only focus on the changes in surface reflection and enhancement in device performance after the nanostructure are employed, while there have been hardly any weatherability tests of thin-film solar cells with surface nanostructures. The weatherability of a thin-film solar cell, however, is one of the most important factors of successful and competitive photovoltaic technology. In this study, nanorod arrays of different sizes were employed as antireflection surface structures in CZTSe solar cells. X-ray diffraction (XRD) and scanning electron microscope (SEM) measurements were used to analyse the crystallinity and morphology of the ZnO nanorod surface structures. Angle-dependent UV-Vis spectrometer has also been used to analyze surface reflection of CZTSe solar cell with nano-rods array antireflection layer at different incident angle. Weatherability tests and hydrophobicity tests were conducted on CZTSe solar cells with different antireflection nanorod structures. Damp-heat tests were carried out to analyse the resistivity of the ZnO nanorod layer to heat and humidity; the changes in optical characteristics and electrical characteristics of the CZTSe solar cells after damp-heat damage were also investigated. Water contact angles (WCAs) on the ZnO nanorod array structures were analysed to reveal their hydrophobicity.

## Results

**Figure 1:**
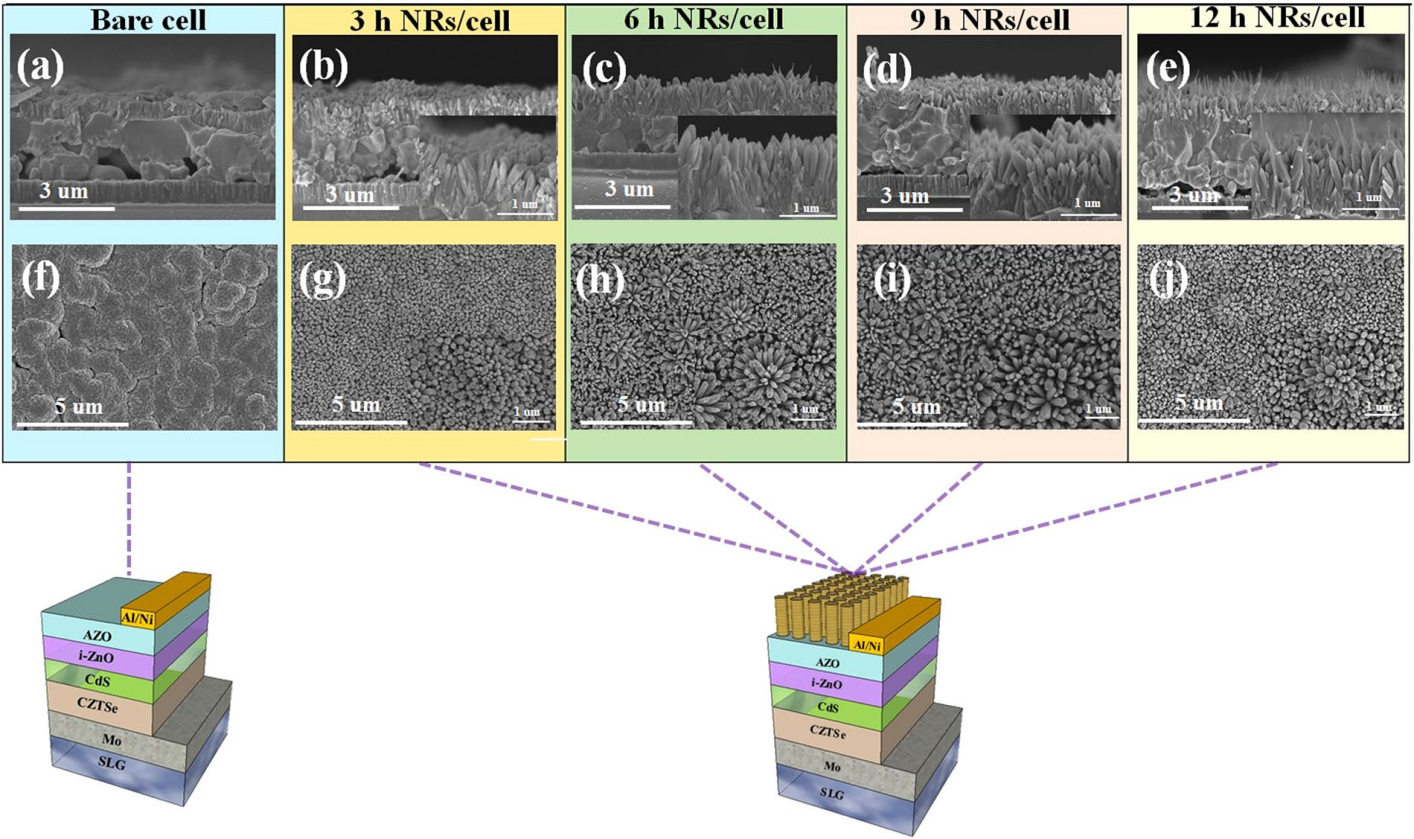
The cross section SEM images of ZnO nanorod arrays on CZTSe solar cells grown with different synthesis time, in which, (**a**) bare device without ZnO nanorod arrays, and nanorods grown for (**b**) three hours, (**c**) six hours, (**d**) nine hours, and (**e**) twelve hours, and top view SEM images of (**f**) bare device without ZnO nanorod arrays, and ZnO nanorod arrays on CZTSe solar cells grown for (**g**) thee hours, (**h**) six hours, (**i**) nine hours, and (**j**) twelve hours.

Figure 1 shows cross-sectional SEM images of ZnO nanorod arrays grown on CZTSe solar cells for different synthesis durations (0, 3, 6, 9, and 12 hr). It can be seen that, between 0 and 9 hr, the height of the ZnO nanorod arrays increased with time, while it decreased to 700 nm as the synthesis duration was extended to 12 hr. The ZnO particles also changed from rod-shaped to needle-like. The profile change and the height reduction ZnO nanorods after 12 h of synthesis occurred because under the synthesis parameters used in this study, the Zn^2+^ ions in Zn(NO_3_)_2_ solution were depleted during the reaction period, which exposed the ZnO nanorods to a stronger alkali environment with higher number of OH^−^ ions, leading to decomposition of the ZnO nanorods in the alkali solution^14^. Figure 1f–j are top-view SEM images of ZnO nanorod arrays on CZTSe solar cells, which respectively shows (f) the bare device without ZnO nanorod arrays, and ZnO nanorod arrays grown for (g) 3 h, (h) 6 h, (i) 9 h, and (j) 12 h. The SEM images in the top row show that the ZnO nanorod arrays became more distinguishable as the synthesis time was extended from 0 to 9 h, while the the ZnO nanorod arrays became irregular after 12 h of synthesis. The diameter of the ZnO nanorod was about 100 nm regardless of the synthesis duration.

The diameter and height of ZnO nanorod arrays used in this study were determined according to the absorption spectral range of the CZTSe solar cell. Considering the intensity distribution over the solar spectrum and absorption range of a CZTSe solar cell, the ZnO nanorod arrays should be focused on antireflection in spectral range of 400–800 nm. The theoretic calculation was based on equation 1 and equation 2, show below^15^,

1 $${\rm{h}}\ge 0.4{\lambda }_{{\rm{longest}}}$$

2 $${\rm{\Lambda }} < \frac{{\lambda }_{{\rm{shortest}}}}{n},$$

where h is the height of ZnO nanorod, λ_longest_ and λ_shortest_ are the maximum and minimum working wavelength for ZnO nanorod arrays, Λ is the period of ZnO nanorods, and n is refractive index of ZnO. According to theoretical calculations, the height of the nanorods should be higher than 500 nm, and the diameter of the nanorods should be smaller than 200 nm; these values were adopted as synthesis parameters in this study. To reveal the light propagation across the interfaces, the distributions of electromagnetic fields within the device structures were simulated by finite-difference time domain (FDTD) analysis (see Supplementary Fig. S1). Details of the simulated parameters are also available (see Supplementary Fig. S1a). The time-averaged TE-polarized electric field intensity distributions, |Ez|, within the solar cells of CZTSe solar cells with different nanorod length are visualized (see Supplementary Fig. S1b). All of the calculated values are normalized to the ones of the excitation source. From results, It can be seen that the field intensities in the CZTSe region are enhanced with ZnO nanorod arrays, and it means the ZnO nanorods not only help light propagate across the interfaces by avoiding the abrupt index transition from air to AZO top layer, but also widen the field distribution within the device by increasing the light scattering on the surface. Strong field intensity between nanorods indicates that the nanorods act as effective scattering centers. Thus the strong scattering within the NRs prohibits the incident light from bouncing back to the free space and enhance the light-trapping effect. Under different incident wavelength illumination, the simulations based on FDTD analysis show that the optical power transmitted into CZTSe absorber layer was significantly enhanced in the length of ZnO nanrods between 600 nm to 900 nm. Experimental observations revealed that ZnO nanorods with a height of 900 nm provided the best antireflection effect, and are in well agreement with the simulations.

**Figure 2:**
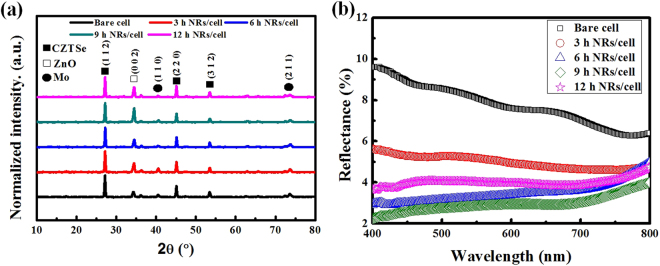
(**a**) XRD spectra and (**b**) reflection spectra of CZTSe solar cells without ZnO nanorod arrays and with ZnO nanorod arrays obtained by different synthesis time.

Figure 2a and b show XRD spectra and reflection spectra of CZTSe solar cells with and without ZnO nanorod arrays prepared over different synthesis durations. The XRD spectra in Fig. 2a show that all of the CZTSe solar cells exhibited diffraction signals from the (112), (220) and (312) crystalline planes of CZTSe and a strong diffraction signal from the Mo substrate. No significant secondary phase can be seen in the XRD spectra. The XRD spectra also show diffraction from the (002) crystalline plane of ZnO, and the diffraction intensity increased from 0 to 9 h for CZTSe solar cells with ZnO nanorods, while the XRD diffraction intensity became weaker for the CZTSe solar cells with ZnO nanorods that were grown for 12 h.

In the reflection spectra shown in Fig. 2b, the average reflectance of CZTSe solar cells without ZnO nanorod arrays was 7.76% in the spectral range of 400–800 nm. For the CZTSe solar cells with ZnO nanorod arrays, the reflectance gradually decreased with time and reached the lowest reflectance of 2.97% for the CZTSe solar cells with ZnO nanorod arrays that were grown for 9 h. However, the reflectance increased to 4.01% for the CZTSe solar cells with ZnO nanorod arrays that were grown for 12 h because the arrays provided a layer with a refractive index between those of AZO and air (the refractive indices gradually increased in the direction from air to AZO). As the height of the ZnO nanorod arrays increased, the change in refractive indices in the ZnO rod layer became smoother, bringing about a more significant antireflection effect. As the height of the ZnO nanorod arrays was reduced when the synthesis duration exceeded 12 h, the change in refractive index became more abrupt, leading to increased reflectance. These observations coincide with the morphology changes shown in the SEM images.

**Figure 3:**
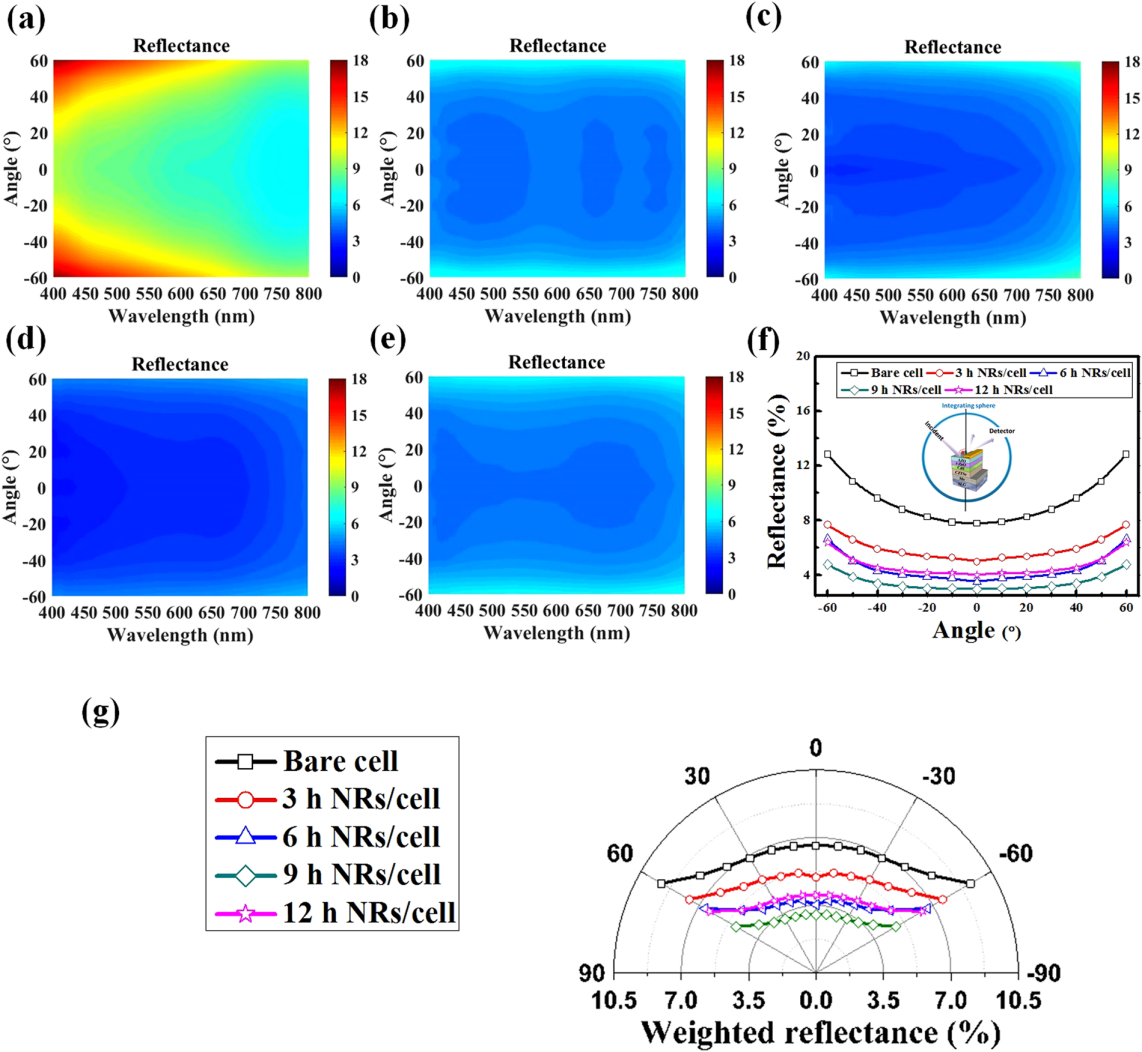
Angle dependent reflection mapping in spectral range of 400 nm to 800 nm of (**a**) CZTSe solar cell without ZnO nanorod arrays, and CZTSe solar cells with ZnO nanorod arrays grown for (**b**) three hours, (**c**) six hours, (**d**) nine hours, and (**e**) twelve hours. (**f**) The average reflectance of CZTSe solar cells with different ZnO nanorod arrays at different angles. (**g**) The weighted reflectance of all cells.

**Figure 4:**
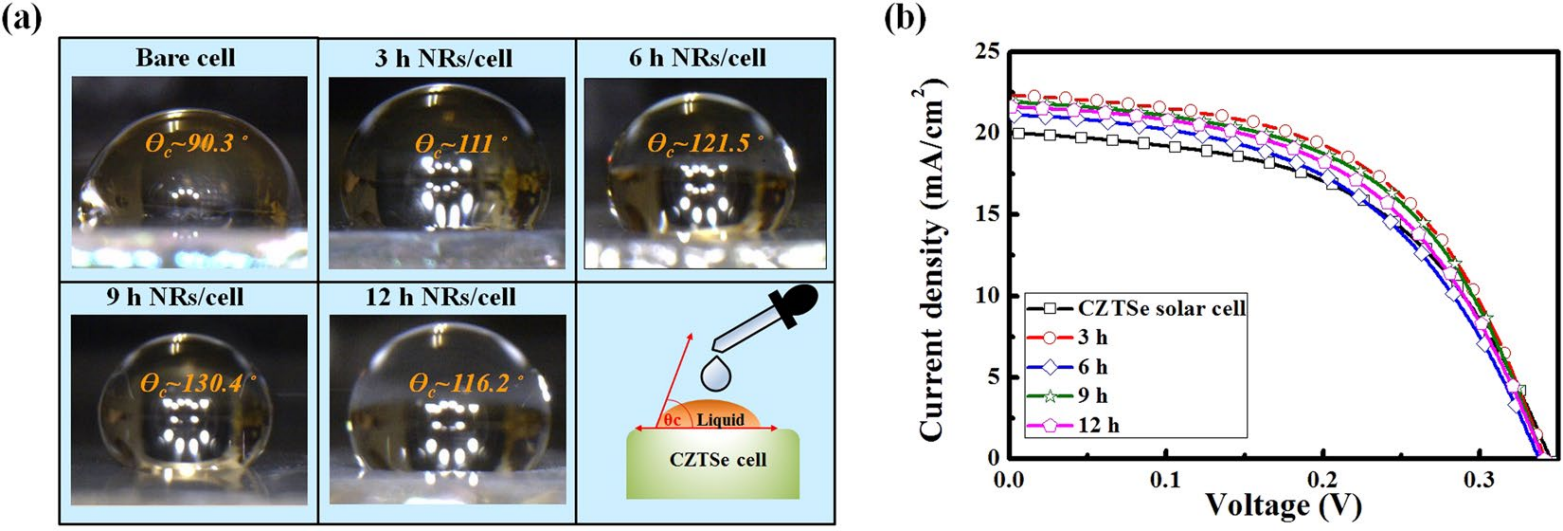
(**a**) Photos of water contact angle tests of CZTSe solar cells with ZnO nanorod arrays grown with different synthesis time, and (**b**) IV curves.

For the nanostructure antireflection scheme designed for solar cells, it is important to take the changing incident angle of solar light into account. Angle-dependent reflection measurements were thus conducted, and the results are shown in Fig. 3a–f. Figures 3a and 4e show the reflectance mapping at different incident angles and different wavelengths for CZTSe solar cells with and without ZnO nanorod arrays prepared over different synthesis durations. The incident angle was varied from −60° to 60°, while spectral range of measurement was 400–800 nm. As shown in Fig. 3a, the CZTSe solar cell without ZnO nanorod arrays showed higher reflectance at large incident angles, while in Fig. 3b–d, the difference in reflectance of light with different incident angles was gradually eliminated as the height of the ZnO nanorod arrays increased. As expected, the difference in reflectance at different incident angles subsequently became higher for the CZTSe solar cell with ZnO nanorod arrays that were grown for 12 h. Figure 3f shows the average reflectance of light at different angles over the spectral range for the measurements. It can be seen that the CZTSe solar cell without ZnO nanorod arrays had an average reflectance of 7.61% of light with zero incident angle, and an average reflectance of 12.81% of light with 60° incident angle, showing a difference of 5.2% in reflectance. For the CZTSe solar cell with ZnO nanorod arrays that were grown for 9 h, the reflectance average at zero incident angle and 60° incident angle decreased to 2.97% and 4.77%, respectively, showing that the difference in average reflectance shrank to 1.8%. These results indicate that the ZnO nanorod arrays successfully provided an omnidirectional antireflection effect to the CZTSe solar cells for light at incident angles ranging from −60° to 60° in the spectral range of 400–800 nm. To accommodate the intensity distribution over the solar spectrum for reflectance enhancement analysis, the solar-spectrum weighted reflectance (SSWR) was calculated using the following equation^16^

3 $${\rm{SSWR}}=\frac{{\int }_{400\,nm}^{800\,nm}R(\lambda ){I}_{{\rm{AM1}}{\rm{.5G}}}(\lambda )d\lambda }{{\int }_{400\,nm}^{800\,nm}{I}_{{\rm{AM1}}{\rm{.5G}}}(\lambda )d\lambda }$$

where R(λ) is the measured reflectance, and I_AM1.5G_ is the photon flux density of the AM1.5 G solar spectrum used for he electrical characterization of CZTSe solar cells in this study. The SSWR values show the percentage of reflected solar light in the range of the solar spectrum considered in this study (400–800 nm). Figure 3g shows the weighted reflectance of the CZTSe solar cells with ZnO nanorod arrays prepared over different synthesis durations. The reflectance of light at all incident angles was lower than that of the bare cell. The SSWR calculations revealed lower values at all angles as the synthesis time of the ZnO nanorod arrays increased, and the value slightly increased for the CZTSe solar cell with ZnO nanorod arrays grown for 12 h. Moreover, the difference between SSWR values obtained at incident angles of 0° and 60° was significant and reached 2.97% for the CZTSe solar cell without ZnO nanorod arrays. For the CZTSe solar cell with ZnO nanorod arrays grown for 9 h, which exhibited the optimum antireflection effect, the difference between SSWR values obtained at incident angles of 0° and 60° was suppressed to 1.78%, demonstrating that the antireflection effect of the ZnO nanorod arrays not only had a broad-band antireflection effect, but was also most effective at the wavelengths with highest intensities in the AM1.5 G solar spectrum, leading to the highly reduced reflectance. This shows that the ZnO nanorod arrays exhibited highly preferable antireflection characteristic for solar-cell applications. It also shows that the omnidirectional antireflection effect of ZnO nanorods can potentially serve as an alternative to the highly expensive solar-tracking system.

Figure 4a shows the photos taken during the hydrophobicity tests of ZnO nanorod arrays grown over different synthesis durations on different CZTSe solar cells. The WCAs are also shown in the photos. The WCA was 90.3° for the CZTSe solar cell without ZnO nanorod arrays. The maximum WCA of 130.4° was obtained on the CZTSe solar cell with ZnO nanorod arrays grown for 9 h, and a WCA of 116.2° was obtained on the CZTSe solar cell with ZnO nanorod arrays grown for 12 h. These results show that the WCA increased as the height of ZnO nanorod arrays increased, indicating that the taller ZnO nanorod arrays possessed higher hydrophobicity. It is known that the AZO window layer can deteriorate in a high-humidity environment owing to the hydrolysis nature of AZO. Therefore, ZnO nanorod arrays can provide perfect protection to prevent damage to the AZO window layer by water drops.

Figure 4b shows the current–voltage (I–V) curves of all CZTSe solar cells. The I–V characteristic parameters are listed together with the WCA values in Table 1. The efficiency of CZTSe solar cells without ZnO nanorod arrays was 3.58%, while efficiencies of 3.79%, 3.97%, 4.08%, and 3.59% were obtained for CZTSe solar cells with ZnO nanorod arrays grown for 3, 6, 9, and 12 h, respectively. No notable changes were observed in the open-circuit voltage (V_OC_). Meanwhile, short-circuit current densities (J_SC_) of 19.91, 21.60, 21.88, 22.22, and 21.10 mA/cm^2^ were obtained for the CZTSe solar cell without ZnO nanorod arrays, and the CZTSe solar cells with ZnO nanorod arrays grown for 3, 6, 9, and 12 h, respectively. The I–V measurements indicate that the efficiency enhancements were mostly brought about by the enhanced J_SC_, while the effect of V_OC_ was irrelevant. Both cell efficiencies and J_SC_ increased as the ZnO nanorod synthesis duration was increased from 0 to 9 h, and the values decreased upon further increase of the synthesis duration to 12 h, which coincide with the changes in SSWR values. In addition, it is noteworthy that both ZnO NRs length and resistance (R_s_) were strongly correlated. As the growth time was increased to 9 hours, the increased length accompanied the decreased resistance. This phenomenon can be ascribed to the imperfect coverage between Al electrodes and bottom Al:ZnO layers with rough surface. During this time-interval, the pores or voids may be filled in with ZnO materials and improve the electricity. Contrary to expectation, longer growth time (12 hours) resulted in decomposition of the ZnO materials in the alkali solution and thus deteriorated the performance. These results indicate that the efficiency enhancement was the result of the antireflection effect of the ZnO nanorod arrays, which led to a maximum J_SC_ enhancement of 11.60% when compared to the J_SC_ values of the CZTSe solar cell without ZnO nanorod arrays and the CZTSe solar cell with ZnO nanorod arrays grown for 9 h.

**Table 1:** IV characteristic parameters, WCAs, SEM, and SSWR values of CZTSe solar cells without ZnO nano-rod arrays, and with different height of ZnO nanorod arrays.

Sample	Bare cell	NRs cell(3 h)	NRs cell (6 h)	NRs cell (9 h)	NRs cell (12 h)
V_oc_(V)	0.36	0.35	0.35	0.35	0.35
J_sc_ (mA/cm^2^)	19.91	21.60	21.88	22.22	21.10
R_s_ (Ω•cm^2^)	4.57	4.42	3.8	3.31	4.47
F.F.	0.51	0.51	0.52	0.53	0.49
Efficiency (%)	3.58	3.79	3.97	4.08	3.59
Angle 0- SSWR (%)	6.49	4.95	3.53	3.01	4.03
WCA (°)	90.3	111	121.5	130.4	116.2
Length (μm)	0	0.4	0.7	0.9	0.7

**Figure 5:**
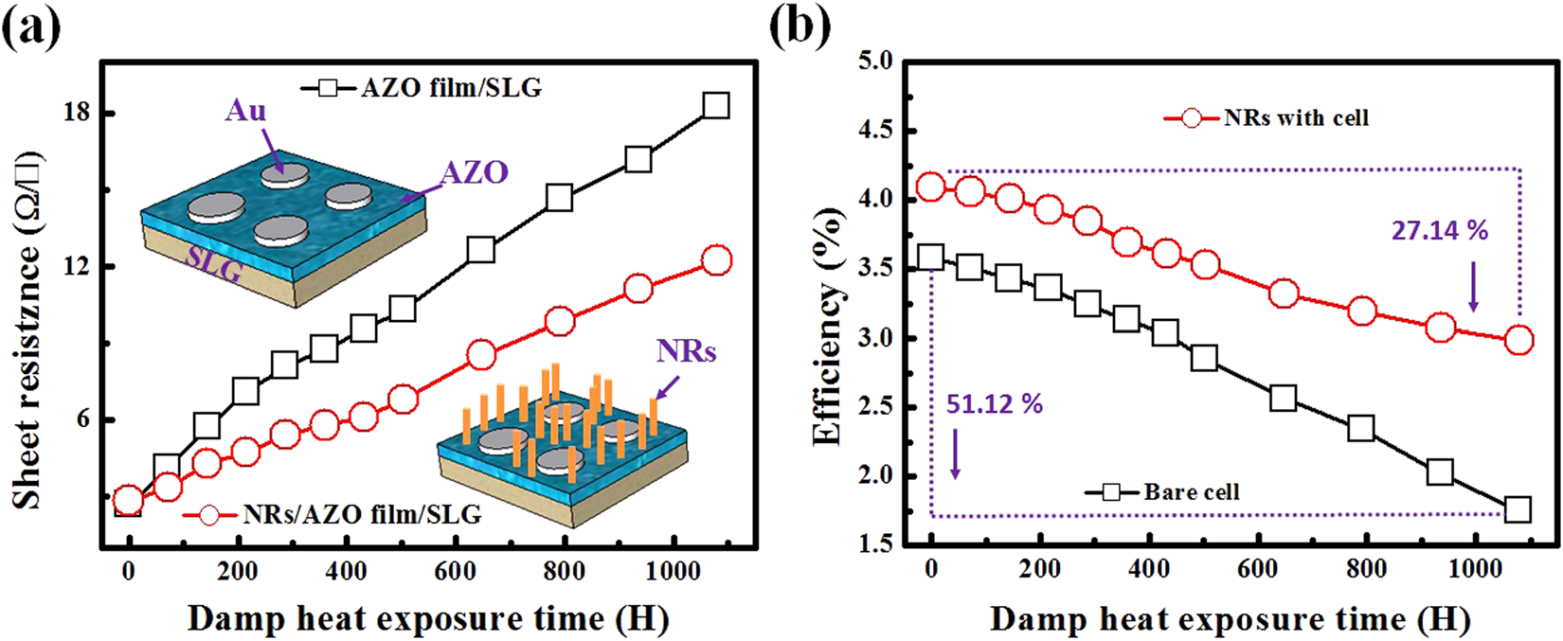
(**a**) Decay of sheet resistance of AZO thin films prepared on soda lime glass with and without ZnO nanorod arrays during damp-heat test. (**b**) Decay of efficiencies of CZTSe solar cells with and without ZnO nano-rod arrays during damp-heat test.

From the quantum efficiency (EQE) measurement shown in Fig. S2 in the Supporting Information, it is evident that the CZTSe solar cells with NRs structures demonstrate an enhanced photoresponse, especially in the wavelength ranging from 500 to 850 nm. This observation confirms that the improvement in J_sc_ of the CZTSe solar cell with NRs structures can be attributed to the better antireflection performance. Weatherability tests were also conducted to examine the resistivity against damp-heat damage of ZnO nanorod arrays on AZO thin films, and the durability of CZTSe solar cells with and without ZnO nanorod arrays. The results are shown in Fig. 5. Figure 5a shows the changes in sheet conductivity of AZO thin films with and without ZnO nanorod arrays during exposure to damp-heat damage. To measure the change in sheet conductivity of the AZO thin films, two AZO thin films were prepared on soda lime glass and both were deposited with Au electrode pads. ZnO nanorod arrays with synthesis time of 9 h were prepared on one of the AZO thin films with Au electrode pads. The two AZO thin films were then placed in a thermostat-controlled chamber with humidity of 85% and temperature of 85 °C for 1000 h. Figure 5a shows that the sheet resistance of the bare AZO thin film without ZnO nanorod arrays increased from 2.67 to 18.29 Ω/square. On the other hand, the sheet resistance of the AZO thin film with ZnO nanorod arrays grown for 9 h increased from 2.82 to 12.22 Ω/square, representing a smaller increase in sheet conductivity. The difference arose the ZnO nanorod arrays had higher hydrophobicity than the AZO thin film, which decreased the density of water drops that directly made contact with the AZO thin film. Moreover, the ZnO nanorod arrays were directly grown on the AZO thin film and served as a protective layer that passivated the hydrolysis characteristic of the AZO thin film. Two CZTSe solar cells were also prepared for damp-heat tests under the same conditions (85% humidity, temperature of 85 °C, dwell time of 1000 h). In particular, one CZTSe solar cell was deposited with ZnO nanorod arrays with synthesis time of 9 h. Figure 5b shows the efficiency decay of the two CZTSe solar cells during the damp-heat test. The efficiency of the CZTSe solar cell without ZnO nanorod arrays decreased from 3.58% to 1.75%, which represents a decrease of 51.12% and the amplitude of change is larger than that of the AZO thin film without ZnO nanorod arrays during damp-heat test (see Fig. 5a). This is due to the existence of water and oxygen, which can lead to delamination or cracking of the interface in CZTSe solar cells^17^, and the formation of higher resistive MoO_x_ layer at CZTSe/Mo interface in high humidity environment^18^. This additional resistive layer has been shown to hinder the transportation of hole carriers and their subsequent collection as the photo-current, thus further destroying the device performance. Similar results were obtained for the CZTSe solar cell with ZnO nanorod arrays and the CZTSe solar cell with an AZO thin film and ZnO nanorod arrays. However, the efficiency of the CZTSe solar cell with ZnO nanorod arrays decreased from 4.09% to 2.98%, showing an amplitude of efficiency decay smaller than that observed for the CZTSe solar cell without ZnO nanorod arrays. This indicates that the ZnO nanorod arrays on AZO provided sustainable protection of the CZTSe solar cell from damp-heat damage. We also conducted to examine the efficiency against open-air treatment of CZTSe solar cells with and without ZnO nanorod arrays. The variation in power conversion efficiency of solar cells with and without ZnO nanorods treated under damp heat and open-air conditions are shown in Fig. S3a in the Supporting Information. The set of solar cell with ZnO nanorod exposed to open-air shows no significant aging effect, whereas the set of solar cell without ZnO nanorod exposed to open-air shows a larger degradation behavior in Fig. S3b in the Supporting Information. Thus the solar cell with ZnO nanorod arrays manifested better environmental stability.

## Discussion

ZnO nanorod arrays were successfully employed as an antireflection layer to enhance the efficiency of CZTSe solar cells. ZnO nanorods of different sizes were studied, and ZnO nanorods with a height of 900 nm provided the best antireflection effect. The average reflectance of the CZTSe solar cell with ZnO nanorods with height of 900 nm decreased from 7.76% of the bare device to 2.97%, while the efficiency increased from 3.58% to 4.08%, showing an efficiency boost of 13.97%. Angle-dependent reflection measurements showed that the antireflection effect of the ZnO nanorod arrays was omnidirectional. According to the results of hydrophobicity tests and weatherability tests, the ZnO nanorod arrays provided a highly hydrophobic surface, which increased the water contact angle from 90.3° to 130.4°, and it imparted high resistance against damp-heat damage to the CZTSe solar cells. In other words, the ZnO nanorod arrays not only served as an omnidirectional antireflection layer, but also as a sustainable protective layer against damp-heat damage. Furthermore, ZnO nanorod arrays can be an effective alternative to conventional solar tracking systems.

## Methods

The CZTSe thin film was prepared by evaporation and post-deposition selenization. First, a Mo layer with thickness of 800 nm was deposited on soda lime glass. This was followed by evaporation of Cu, Zn, and Sn thin films (99.999% purity) as precursor layers. The sample was then sent to a vacuum chamber for post-deposition selenization. Selenium gas was fed into the chamber by thermally evaporating solid bulk selenium in a quartz holder and purging the chamber with Ar gas. The peak selenization temperature was 500 °C. After the CZTSe thin film was prepared on the Mo thin film, a CdS buffer layer with thickness of 50 nm was deposited onto the CIGS thin film by chemical bath deposition (CBD). After that, an intrinsic ZnO layer (i-ZnO) with thickness of 50 nm and an Al-doped ZnO (Al:ZnO) layer with thickness of 300 nm were deposited on top as window layers. The fabrication of the CZTSe solar cell was complete after deposition of an Al grid with thickness of 1 µm and area of 0.18 cm^2^ as the top electrode. The efficiencies of CZTSe solar cells were obtained by excluding the area of the top electrode grids from the total illuminated device area. Five CZTSe solar cells were prepared with ZnO nanorod arrays of different sizes. A reference CZTSe solar cell without ZnO nanorod arrays was prepared for comparison.

**Figure 6:**
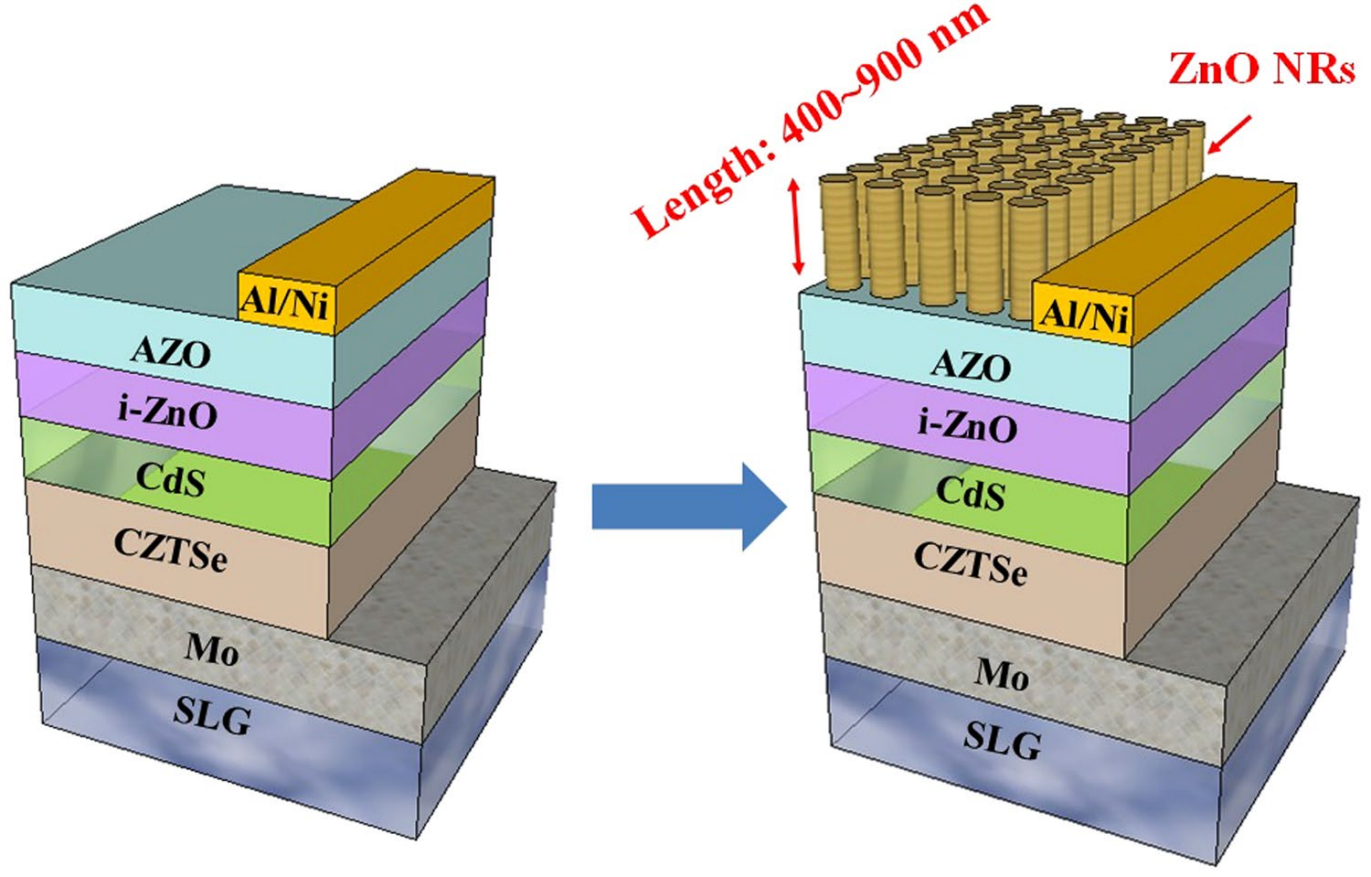
Schematic illustration of CZTSe solar cells and ZnO nanorod arrays. The size of ZnO were denoted.

The ZnO nanorod arrays of different sizes were prepared on different CZTSe solar cells by the chemical solution method. First, zinc nitrate [Zn(NO_3_)_2_6H_2_O, purchased from Aldrich] and hexamethylenetetramine (C_6_H_12_N_4_, HMT, purchased from Aldrich) was diluted with deionized (DI) water to 0.02 M. The ZnO nanorod arrays of were prepared in a thermostat-controlled chamber at 90 °C. Depending on the desired size of the nanorods, they were kept in the chamber for differnet periods of time (3–12 h). The structure is schematically illustrated in Fig. 6. The synthesis pathway is show below:

4 $${\mathrm{Zn}(\mathrm{NO}}_{3}{)}_{2}\to {{\rm{Zn}}}^{2+}+2{{\rm{NO}}}_{3}^{-}$$

5 $${{\rm{C}}}_{6}{{\rm{H}}}_{12}{{\rm{N}}}_{4}+6{{\rm{H}}}_{2}{\rm{O}}\to 6{\rm{HCHO}}+4{{\rm{NH}}}_{3}$$

6 $${{\rm{NH}}}_{3}+{{\rm{H}}}_{2}{\rm{O}}\to {{\rm{NH}}}_{4}^{+}+{{\rm{OH}}}^{-}$$

7 $${{\rm{Zn}}}^{2+}+2{{\rm{OH}}}^{-}\to {\mathrm{Zn}(\mathrm{OH})}_{2}$$

8 $${\mathrm{Zn}(\mathrm{OH})}_{2}\mathop{\to }\limits_{{\rm{heat}}}{\rm{ZnO}}+{{\rm{H}}}_{2}{\rm{O}}$$

The morphology of the CZTSe solar cells with nanorod arrays was characterized by field-emission scanning electron microscope (FE-SEM), while the reflectance of the devices with nanorod arrays was determined from UV–Vis measurements. To characterize the performance of the ZnO nanorods on the CZTSe solar cells, the reflection spectra and angle-dependent reflectance of the samples were measured using a UV-Vis spectrophotometer with a 15 cm-radius integrating sphere at wavelengths ranging from 400 to 800 nm and incident angle from −60° to 60°. A solar simulator was used for electrical characterization of the CZTSe solar cells. A drop of water was placed on the sample surface and the image of this drop was investigated with a contact angle meter (G-1 Goniometer, Erma Optical works, Ltd., Tokyo, Japan) at room temperature. Water contact angles and angle-dependent reflections were measured to obtain the hydrophobicity and omni-directional optical properties of the CZTSe solar cells with nanorod arrays. Damp-heat tests were also conducted at a temperature of 85 °C and humidity of 85% to obtain the weatherability of the CZTSe solar cells with ZnO nanorod arrays. The efficiency decay rate of the cells was obtained by analysing the results.

## Electronic supplementary material

Supplementary Information
