# Supplementary material for: Enhanced omnidirectional and weatherability of Cu2ZnSnSe4 solar cells with ZnO functional nanorod arrays
Source: Sci Rep. 2017 Nov 2;7:14927. doi: 10.1038/s41598-017-14899-4 (PMC5668425; doi:10.1038/s41598-017-14899-4)
Supplement: Supplementary file 1 — Supplementary Information [file 41598_2017_14899_MOESM1_ESM.pdf]

Supplementary Information

Enhanced omnidirectional and weatherability of $Cu_2ZnSnSe_4$ solar cells with ZnO functional nanorod arrays

Fang-I Lai[1,2+], Jui-Fu Yang[1,3+], Wei-Xiang Liao[1], Shou-Yi Kuo [3,4*]

[1] Department of Photonics Engineering, Yuan-Ze University, 135 Yuan-Tung Road, Chung-Li, 320, Taiwan.

[2] Advanced Optoelectronic Technology Center, National Cheng-Kung University, Tainan, 701, Taiwan,

[3] Department of Electronic Engineering, Chang Gung University, 259 Wen-Hwa 1st Road, Kwei-Shan, Tao-Yuan 333, Taiwan.

[4] Department of Nuclear Medicine, Chang Gung Memorial Hospital, No.5, Fuxing Street, Kwei-Shan, Tao-Yuan 333, Taiwan

*Corresponding author: sykuo@mail.cgu.edu.tw

+these authors contributed equally to this work

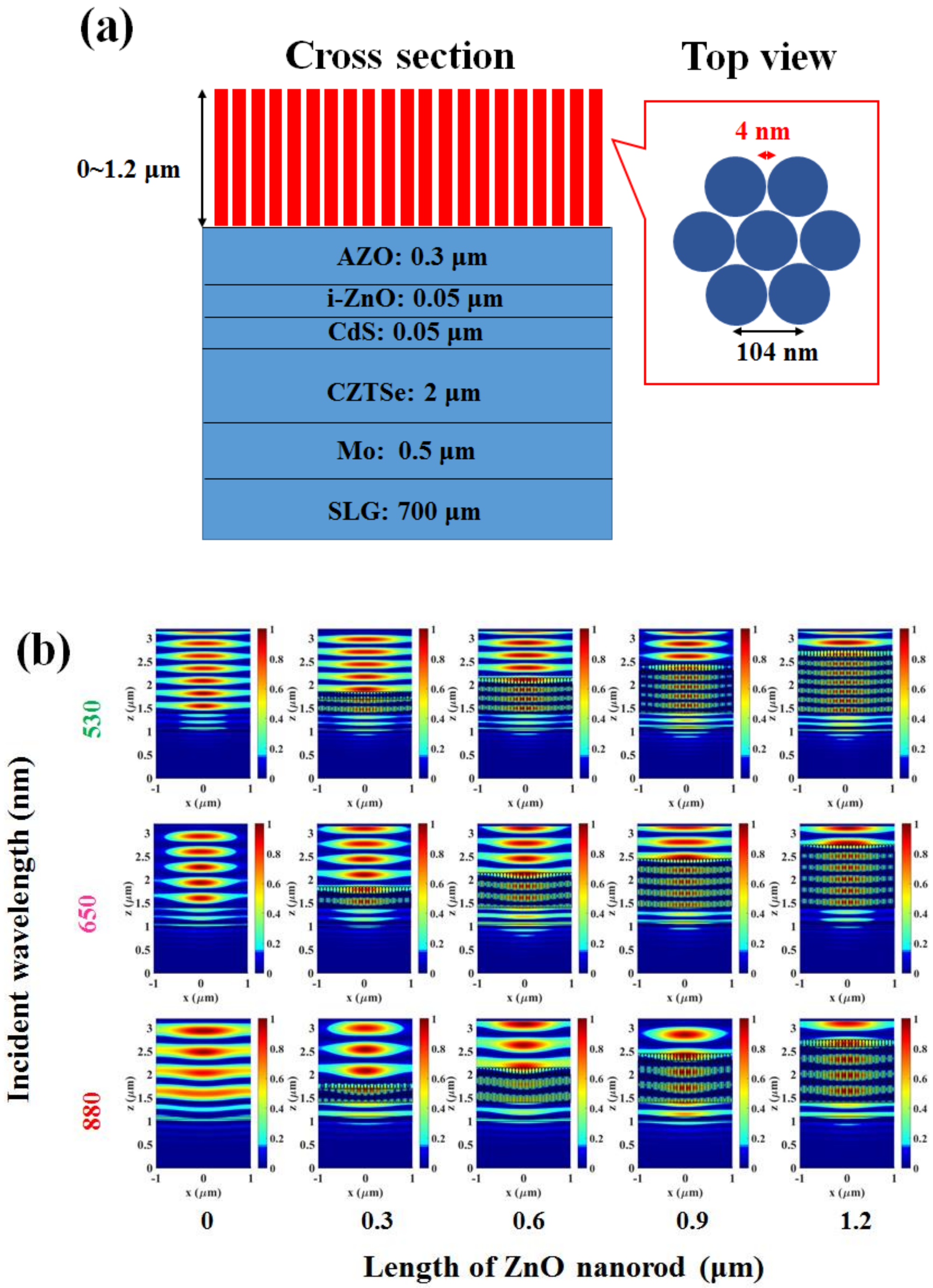

Figure S1 (a) Schematic diagram of the CZTSe solar cell with ZnO nanorod (NRs) arrays for FDTD simulations. Dimensions of the nanorods were the average values determined by SEM images shown in Fig. 1(a)-2(e) in the manuscript. According to the SEM top-view images, the period of ZnO nanorod arrays is ~ 104 nm. (b) Time-averaged and normalized TE electric field distribution, |Ez|, simulated by FDTD analysis within the CZTSe solar cells containing with different ZnO nanorods length and incident wavelength.

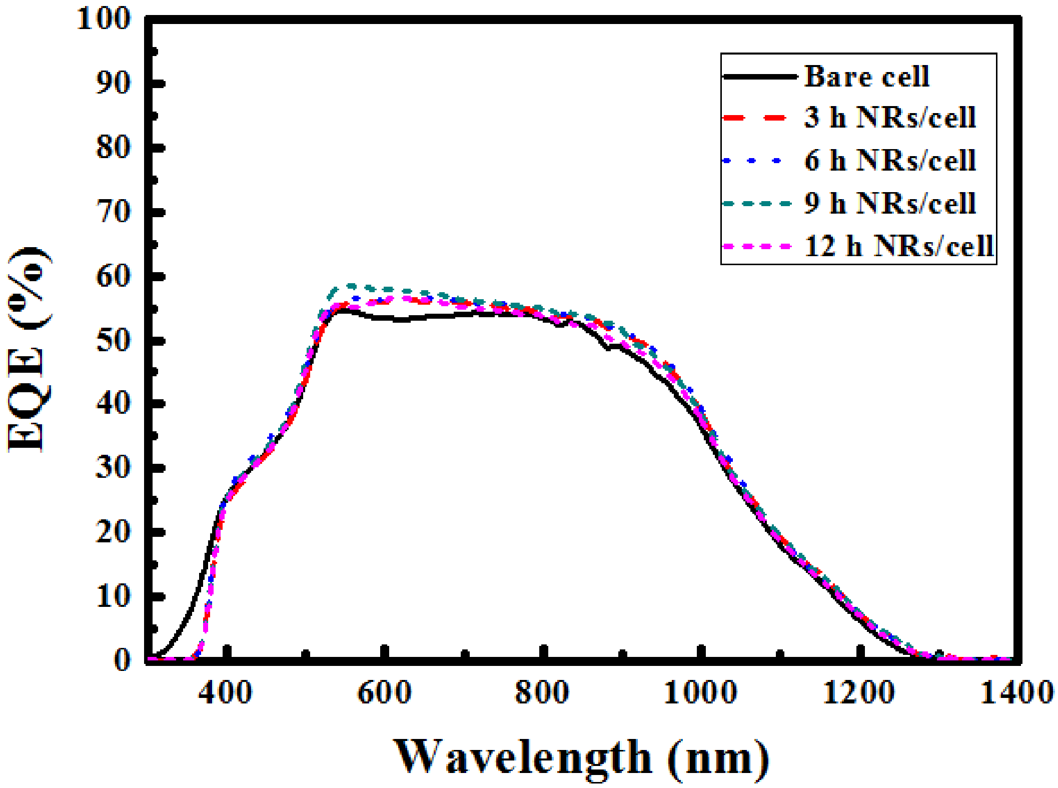

Figure S2 EQE spectra of ZnO nanorod arrays on CZTSe solar cells grown with different duration.

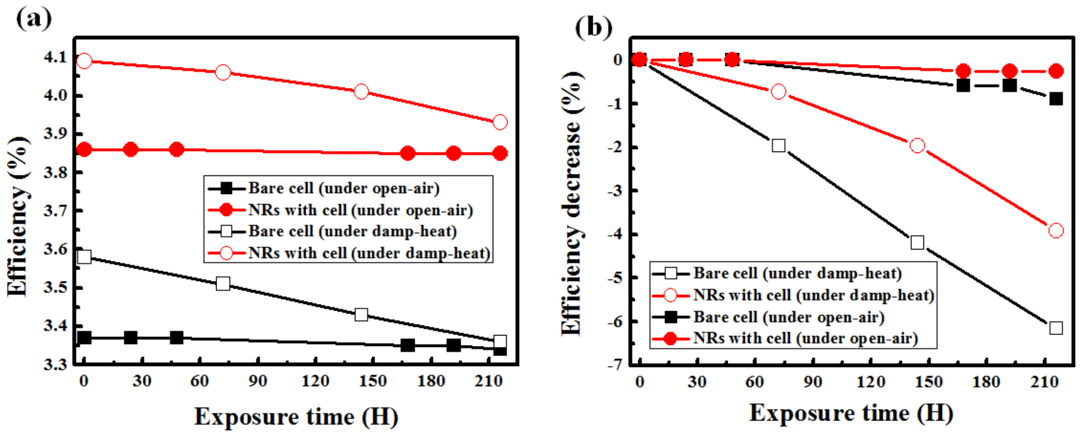

Figure S3 (a) Variation of efficiencies of CZTSe solar cells with and without ZnO nanorod arrays under long-term damp-heat and open-air treatments. (b) Long-term characterization of the CZTSe solar cells with/without ZnO nanorods structure.
